# Infant Barlow’s Disease in Association with Atrial Septal Defect

**DOI:** 10.21470/1678-9741-2023-0278

**Published:** 2024-05-13

**Authors:** Isaac Azevedo Silva, Larissa Ales Leite Matos, Carolina Sant’Anna, Ulisses Alexandre Croti

**Affiliations:** 1 CardioPedBrasil® - Centro do Coração da Criança at Hospital da Criança e Maternidade São José do Rio Preto, São Paulo, Brazil (FUNFARME/FAMERP)

**Keywords:** Mitral Valve Insufficiency, Mitral Valve Annuloplasty, Congenital Heart Defect, Atrial Septal Defect, Thoracic Surgery

## Abstract

Clinical data: Female, seven years old, referred to our service complaining about congestive heart failure symptoms due to mitral valve regurgitation and atrial septal defect. Technical description: Echocardiographic findings compatible with Barlow’s disease and atrial septal defect, ostium secundum type. Operation: She was submitted to mitral valvuloplasty with chordal shortening and prosthetic posterior ring (Gregori-Braile®) along with patch atrioseptoplasty. Comments: Mitral valve regurgitation is a rare congenital heart disease and Barlow’s disease is probably rarer. Mitral valve repair is the treatment of choice.

## CASE PRESENTATION

### Clinical Data

**Table:** 

Abbreviations, Acronyms & Symbols	
ASD	= Atrial septal defect
BD	= Barlow’s disease
MV	= Mitral valve

Female, seven years old, weighting 23.9 kg, height 1.25 m, with a history of long-term exertional dyspnea, referred to our hospital due to progressive worsening of symptoms. She was diagnosed at seven years old with atrial septal defect (ASD) and mitral valve (MV) prolapse and regurgitation, and since then, she has been on medical therapy.

On admission, the patient was New York Heart Association class II for congestive heart failure.

## TECHNICAL DESCRIPTION

### Chest Radiography

**Fig. 1:**
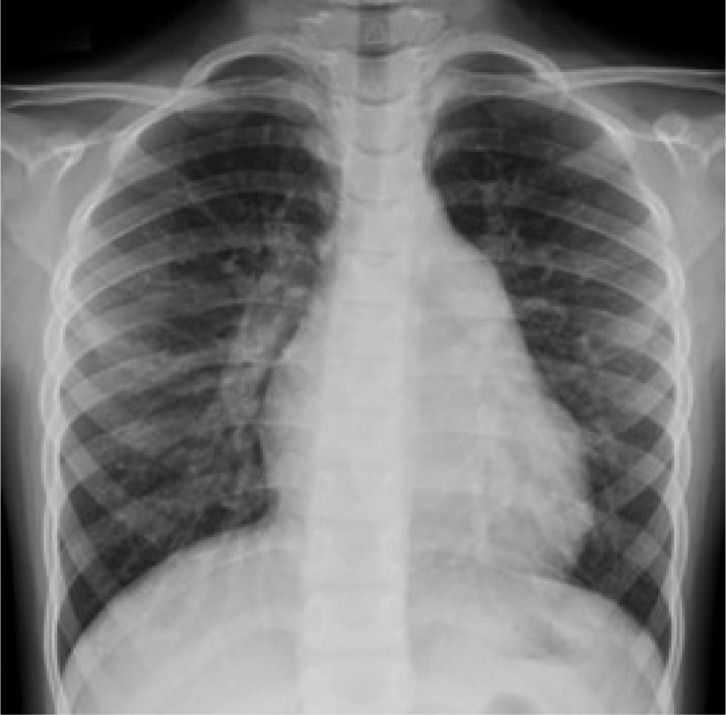
Chest radiography with prominent pulmonary vascular markings and mild cardiomegaly.

Chest radiography shows increased pulmonary vascular markings, no pulmonary edema, and mild cardiomegaly with cardiothoracic ratio of 0.55 (Figure 1).

### Electrocardiography

Sinus rhythm (S QRS 106°), PR interval of 155 ms, QRS of 78 ms, QTc of 432 ms, heart rate of 88 bpm, and left atrial enlargement.

### Transesophageal Echocardiography

*Situs solitus* in levocardia, usual venoatrial, atrioventricular, and ventriculoarterial connections.

**Fig. 2:**
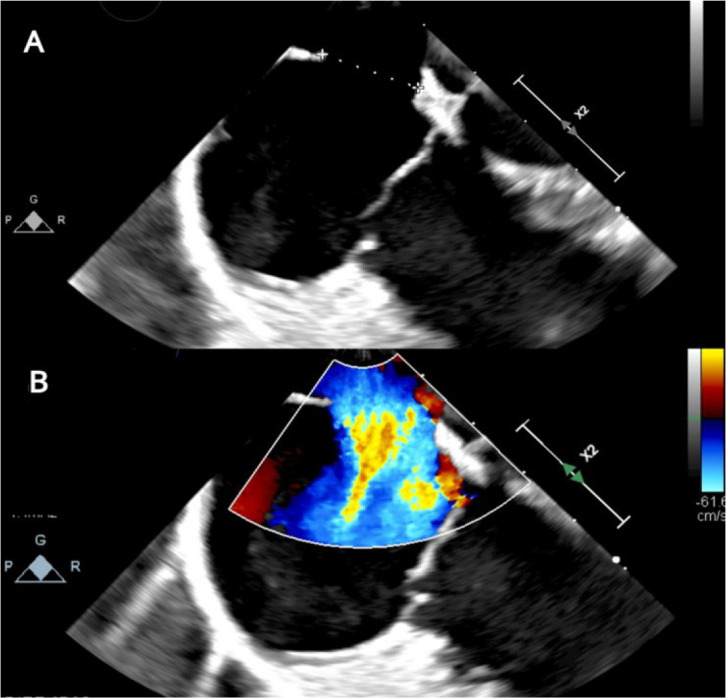
Apical four-chamber transesophageal echocardiogram, two-dimensional view. A) Imaging with probe rotated toward right-sided structures, showing dropout in mid-septum between the left atrium and right atrium consistent with ostium secundum atrial septal defect (ASD). B) Addition of color Doppler shows left-to-right shunt through the ASD.

**Fig. 3:**
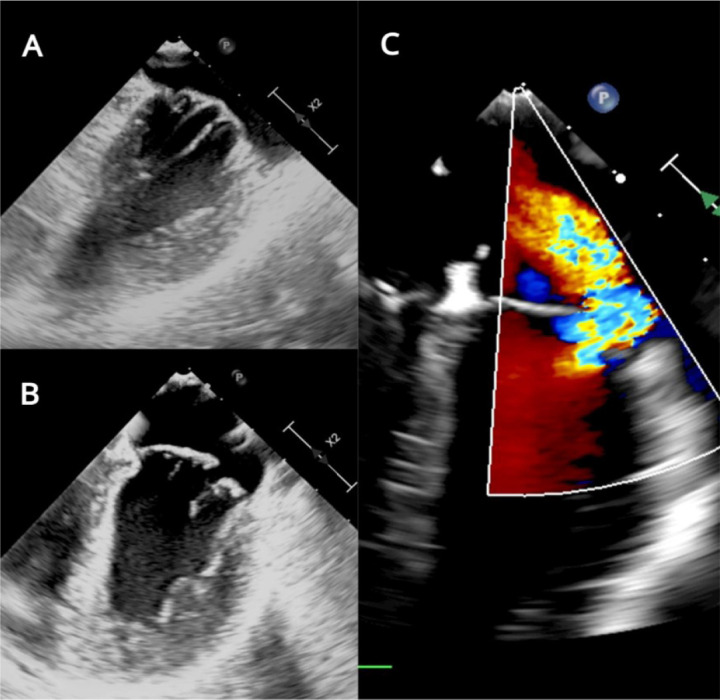
Transesophageal echocardiogram, two-dimensional view of mitral valve. A) Prolapsed, thickened, and elongated chordae tendineae. B) Failure of coaptation and billowing anterior segments (A2 and A3) and posterior scallops (P2 and P3). C) Addition of color Doppler shows severe mitral regurgitation.

**Fig. 4:**
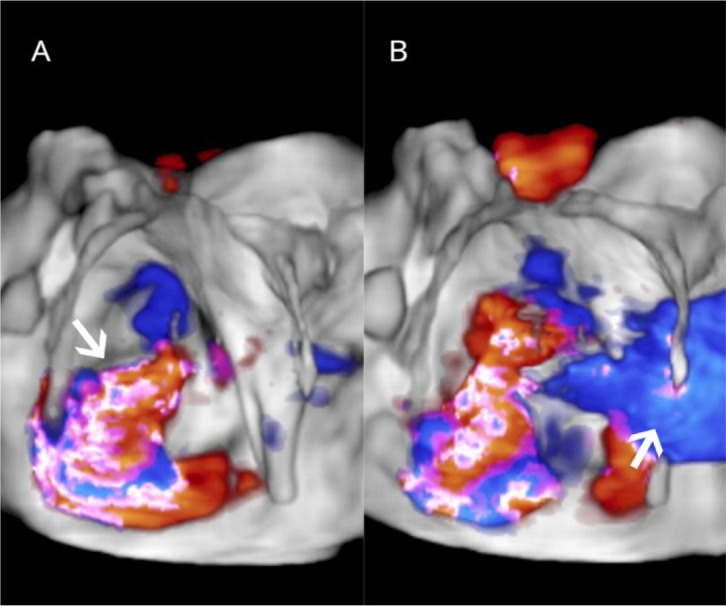
Three-dimensional echocardiogram image of the mitral valve from the left atrial perspective. Color Doppler demonstrates valve regurgitation (arrow) (A) and left-to-right shunt through the atrial septal defect (arrow) (B).

Presence of *ostium secundum* ASD of 14 mm with left to right shunt (Figure 2). Normal biventricular ejection fraction, enlargement of right cardiac chambers and left atrium, and noticeable MV insufficiency (Figures 3C, 4A, 4B).

**Fig. 5:**
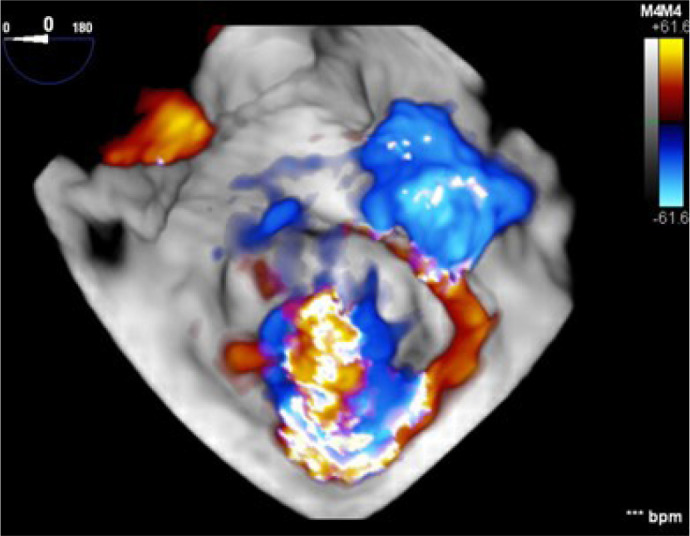
Three-dimensional image from the left atrial perspective demonstrating features typical of Barlow’s disease with severe mitral regurgitation, annular dilation, leaflet redundancy, and multisegmental prolapsing/billowing mitral valve components.

MV features included annular dilation, leaflet redundancy, with failure of coaptation between A2-A3 and P2-P3, associated with multisegmental prolapsing/billowing MV components, and thickened, elongated *chordae tendineae*, typical of Barlow’s disease (BD) (Figures 3A, 3B, 5).

No abnormalities in other valves. Additional findings were unremarkable.

### Operation

**Fig. 6:**
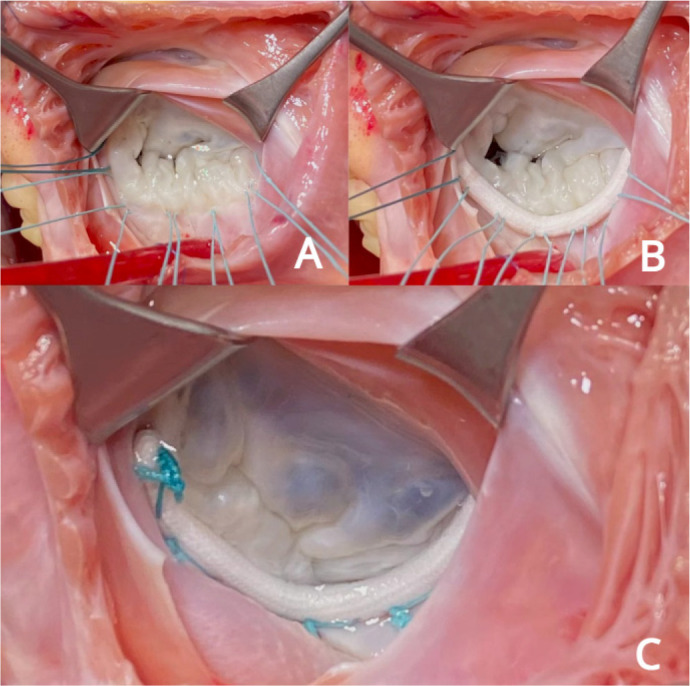
Surgical aspect of the mitral valve from atrial view. A) After atriotomy, with ring repair sutures in place (note diffuse thickening of the anterior leaflet with failure of coaptation between A2-A3 and P2-P3). B) Imaging after implantation of a rigid 26 mm posterior ring (Gregori-Braile®). C) Final aspect with ring in place and proper coaptation of the leaflets after testing with saline solution (NaCl 0.9%).

After median sternotomy, cardiopulmonary bypass was established with bicaval and ascending aorta cannulation. A single dose of antegrade cold crystalloid cardioplegia, Custodiol-HTK® (GmbH, Bensheim, Germany), was given. Through left atriotomy in interatrial groove, the MV was evaluated confirming echocardiographic findings (Figure 6A).

MV repair was performed by chordal shortening and implantation of rigid 26 mm posterior ring (Gregori-Braile®, Braile Biomédica, São José do Rio Preto/São Paulo, Brazil) (Figures 6B, 6C). The *ostium secundum* ASD was closed with bovine pericardial patch.

Transesophageal echocardiogram was performed after cardiopulmonary bypass weaning, showing minimal MV regurgitation, no residual shunts, and preserved biventricular function.

## COMMENT

Congenital MV lesions are a rare and particularly degenerative MV disease. An echocardiographic study detected MV congenital malformations in approximately 0.5% of 13,400 subjects^[1,2]^. There is no clear information about the incidence of BD in infants and children. Indeed, the diagnosis of BD, even in adults, has been raising concerns, as shown by Carpentier’s group^[3]^.

Histologically, normal MV tissue consists of three layers. The atrialis, on the atrial side, is rich in elastic fibers, providing elasticity to the valve. The spongiosa, in the middle, is made of glycosaminoglycans and proteoglycans, supplying flexibility to the valve, absorbing vibrations. And the fibrosa, on the ventricular side, is the thickest part of the leaflet and is rich in collagen fibers, providing tensile strength to the valve^[4]^.

In BD, the organization of the three layers is disrupted. Collagen and elastin fibers are fragmented, and the spongiosa layer expands due to accumulation of proteoglycans, characteristic of myxomatous degeneration, and infiltrates the fibrosa layer^[3]^.

On echocardiography, BD is characterized by a diffuse, leaflet redundancy, with bileaflet prolapse or prolapse of multiple segments. Valve leaflets are also often thickened (> 3 mm) as measured in diastole using the M-mode. Chordae are also frequently thickened and chordal elongation is more common than chordal rupture^[5]^.

MV regurgitation was the classical manifestation of BD, and repair with a prosthetic posterior ring has been proved to allow better outcomes than with complete rings^[6]^.

In this presented case, echocardiographic landmarks of BD were found and confirmed on surgical exploration. A no-resection MV repair was successfully achieved through chordal shortening and prosthetic posterior annulus approach along with ASD closure.
